# Supplementary material for: An internet-delivered acceptance and commitment therapy program for anxious affect, depression, and wellbeing: A randomized, parallel, two-group, waitlist-controlled trial in a Middle Eastern sample of college students
Source: PLoS One. 2024 Dec 5;19(12):e0313243. doi: 10.1371/journal.pone.0313243 (PMC11620599; doi:10.1371/journal.pone.0313243)
Supplement: S1 File — Complete description of the content for each module contained in the program. (DOCX) [file pone.0313243.s001.docx]

**Supporting information. Description of the program’s content.**

*Module 1:* The ACT program commenced with an introductory module which briefed participants about the program, explained how the training provides general psychological and behavioural skills that may be used in all facets of life. Moreover, it discussed how ACT is associated with decreased stress and elevated well-being [1]. Participants were presented with a two-skills diagram, mindfulness and values-based action, as the combination of these two skills has regularly been shown to increase individuals’ well-being and behavioural effectiveness [1]. The first mindfulness activity was the ‘raisin exercise’, in which participants were required to eat a raisin with heightened mindful awareness. Instructions were provided to increase awareness in relation to the sensations associated with observing, touching, eating, and swallowing the raisin. This exercise promotes the enhancement of the capacity to switch off from automatic pilot and be more present in the moment [2]. The second mindfulness activity presented was a ‘breath and body’ exercise which also elevates the enhancement of full awareness of the body. Participants were required to close their eyes and follow simple instructions to focus on bodily sensations. The second half of the session was spent on the companion skill, value-based action. Value-based actions are defined as activities that enable the individual to manifest a value, which was frequently referred to throughout the training program as ‘bringing a value to life’. A compass metaphor was used to explain this phenomenon whereby values direct our behaviours just like a compass does [2]. Lastly, participants were asked to complete the Survey of Life Principles, which helps in defining the values that the participant wants to translate to actions [3]. Participants were requested to define 2 to 3 values that they wished to work on in preparation for the second module.

*Module 2:* The second module, titled ‘Values, Goals and Actions’, was designed to equip the participants with a range of skills aimed at fostering their mindfulness and facilitating value-based actions. According to ACT, values are described as deliberate concepts that connect to patterns of action that provide individuals with a sense of purpose and guidance for their behaviour over long time frames [4]. The module was divided into six subsections, values and their types, values, the goals and action worksheet, the passengers on a bus metaphor, the two sheets of paper activity, a body and breathe exercise, and a homework assignment. The first section defined values, highlighted their importance, and explained the various types of values. The section was designed to remind participants of some of the ideas they had learned in the first module as well as emphasise the role of values in the upcoming exercise titled ‘Values, Goals and Actions’. In the second section, participants were presented with two handouts extracted from the Flaxman et al. manual [2]. The handouts were used to assist participants to define and summarise their values in simple terms, to translate them into more specific goals, and to mindfully identify the values-based actions that they intend to perform in the coming week. The third section explained the concept of ‘internal barriers’. The ‘Passengers on the Bus’ metaphor was used to illustrate how thoughts, feelings, moods, and other internal states influence our pursuit of value-based action [5]. In this metaphor, participants are asked to visualize themselves as bus drivers with their thoughts and emotions as passengers. Through this visualization, they practice observing these thoughts and emotions without judgment, recognizing their transient nature, and realizing their capacity to steer their lives in accordance with their values. This exercise promotes mindfulness and the use of defusion techniques to distance themselves from distressing thoughts. The fourth section included the ‘two sheets on a paper’ technique specifically to help participants develop the ability to pursue value-based actions despite the presence of unhelpful thoughts, moods, emotions, and internal states [2]. Participants are asked to use two sheets of paper, on one side they would write down unhelpful thoughts, beliefs, and moods, while on the other side, they identify helpful values. The fifth section included Body and Breath Exercise that was previously introduced in the first module. The sixth and final section asked participants to complete a homework task to record any "unhelpful" thoughts that hinder their effectiveness or their pursuit of personally valued actions and goals. This exercise encourages self-awareness and the identification of thought patterns that may require attention.

*Module 3:* The third module, titled ‘Defusion’, was designed to entrain in participants the skill of how to separate thoughts from actions. Defusion entails educating individuals to separate thoughts from their literal meaning and to understand that they are more than just the sum of their thoughts, thereby preventing harmful cognitions from having an undue influence on their behaviour [6]. The presentation was divided into five sections, commencing with an introduction to defusion, the concept’s definition, and illustrative examples. The concept of cognitive fusion was also introduced and was contrasted with defusion. In the second part of the presentation, participants were asked to perform the ‘thoughts on a screen’ activity which required them to imagine their thoughts being projected onto a screen. This is similar to the ‘watching thoughts on clouds’ activity which was also included in the following module [7]. The objective of this activity was to help participants learn to be a dispassionate observer, noticing their passing thoughts and for participants to experience a shift from being engrossed in the content of their thoughts to simply observing their thoughts. The following exercise titled ‘Cartoon Voices Techniques’ builds on the previous activity as the participants were asked to identify some of the most unhelpful thoughts in the last exercise and take a moment to notice its impact, after which, a list of well-known cartoon characters (such as Shrek, Homer Simpson, etc.) with easily identifiable voices were presented with their respective images and audio clips. Here, the participants were asked to close their eyes and imagine the previously identified unhelpful thoughts in the voice of a cartoon character of their choice. Then, they were required to open their eyes and experience the thought in its original form and notice its impact. The aim of this exercise was to induce a change in the relationship between the participants and their unhelpful cognitions, allowing them to take negative thoughts less seriously than before. Additionally, it also served the purpose of adding humour to this module which also has a defusive role to play [2]. As the participants at this point would already have identified negative thoughts, the next exercise, ‘Labelling the Mind’, required them to identify a common theme in their unhelpful cognitions and use it to coin a nickname for their mind [2]. Examples for nicknames related to negative thoughts were provided to help the participants. The aim was to facilitate awareness of thoughts and prevent negative cognitions from interfering with value-based action. Finally, as homework for the week, participants were tasked to perform for five days, the ‘Breath and Body’ exercise which was previously performed in the first session.

*Module 4:* The final module aimed to consolidate the skills that had been entrained in the previous sessions and assist participants to engage in increasingly more value-guided actions in their lives. The session consisted of two mindfulness exercises, ‘Clouds Floating’ and the ‘Resilient Observer’, as well as revisiting the ‘Values, Goals and Actions’ exercise from session 2, and concluding with recommendations for continued progress and a reflection on the program in its entirety. ‘Clouds Floating’ was offered as an alternative to the ‘Thoughts on a Blank Screen’ exercise from session 3 and aimed to assist participants develop mindful awareness of their thoughts by imagining their thoughts as if they are placed on clouds drifting across the sky [8]. ‘Resilient Observer’ promotes awareness of the body with all its sensations, as well as the perspective of being the observer of bodily sensations [2]. While thoughts, feelings, and sensations are constantly changing, the observing self is a constant and unchanging aspect of our psychological experience [7]. Participants were then asked to revisit the ‘Values, Goals and Actions’ worksheet that they had seen in session 2. Defining values is not a one-off exercise, and therefore, participants were encouraged to complete the worksheet once again for a different area of their life than the one they chose in session 2. With regard to recommendations for continued progress, participants were encouraged to engage mindfully in three value-based actions per week, define their values, identify a series of more concrete goals and actions that will help to bring their values to life, practice engaging in personally valued actions even when unhelpful or difficult thoughts and feelings have emerged, and lastly, continue practicing mindfulness of the body and breath three times per week. At the end of the session, participants were requested to share their experience, and the impact that the training had on their academic and personal lives.

**References**

1. Wersebe, H., et al., *The link between stress, well-being, and psychological flexibility during an Acceptance and Commitment Therapy self-help intervention.* Int J Clin Health Psychol, 2018. **18**(1): p. 60-68.

2. Flaxman, P.E., Bond, F.W., & Livheim, F., *The mindful and effective employee: An acceptance and commitment therapy training manual for improving well-being and performance.* . 2013, Oakland, CA: New Harbinger.

3. Ciarrochi, J., & Bailey, A., *A CBT-Practitioner's Guide to ACT: How to Bridge the Gap Between Cognitive Behavioral Therapy and Acceptance and Commitment Therapy.* 2008, Oakland, CA.: New Harbinger Publications.

4. Dahl, J., *Valuing in ACT.* Current Opinion in Psychology, 2015. **2**: p. 43-46.

5. Hayes, S.C., et al., *Acceptance and commitment therapy: model, processes and outcomes.* Behav Res Ther, 2006. **44**(1): p. 1-25.

6. Healy, H.-A., et al., *An Experimental Test of a Cognitive Defusion Exercise: Coping With Negative and Positive Self-Statements.* The Psychological Record, 2008. **58**(4): p. 623-640.

7. Hayes, S.C., & Smith, S., *Get out of your mind and into your life: The new Acceptance and Commitment Therapy.* 2005, Oakland, CA: New Harbinger.

8. Hallis, L., et al., *Combining Cognitive Therapy With Acceptance and Commitment Therapy for Depression: A Group Therapy Feasibility Study.* J Cogn Psychother, 2017. **31**(3): p. 171-190.
