# Supplementary material for: An internet-delivered acceptance and commitment therapy program for anxious affect, depression, and wellbeing: A randomized, parallel, two-group, waitlist-controlled trial in a Middle Eastern sample of college students
Source: PLoS One. 2024 Dec 5;19(12):e0313243. doi: 10.1371/journal.pone.0313243 (PMC11620599; doi:10.1371/journal.pone.0313243)
Supplement: Attachment — Submitted filename: Response to reviewers ACT PLOS ONE.docx [file pone.0313243.s003.docx]

**Response to reviewers**

PONE-D-24-06315R1. An internet-delivered acceptance and commitment therapy program for anxious affect, depression, and wellbeing: A randomized, parallel, two-group, waitlist-controlled trial in a Middle Eastern sample of college students

1. Given that, not all the measures employed in this study make provision for the use of cut-off scores, and indeed we have not used the cut-off scores in our principal analyses, this sentence describing the cut-off scores for the GAD-7 has been removed from the Method. Thus, no further analyses using them have been conducted or reported.
2. A power calculation was indeed computed. This is now included in the manuscript as a separate subheading in the Materials and Method section.
3. The method employed to execute multiple imputation is now described in a few additional sentences.
4. A sentence has been added stating that attrition was not related to baseline scores on any of the measures. P17, para 1.
5. Percentage values have been added – table 2.
6. Ranges for each instrument have been added to table 2.
7. No between-group differences at pre-intervention were evident. An additional subheading has been added to the Results describing the results of these comparative baseline analyses.
8. The adjusted between-group post-treatment differences and surrounding confidence intervals have been added to table 2.
9. We made the determination based on an examination of the data distributions that data did not significant deviate from normal and this proceeded with parametric analyses without a consideration of further bootstrapping.
10. Further post-hoc analyses were computed for all the primary variables which emerged as statistically significant in the initial analyses – for the GAD, SIAS, and WEMWS. We have added textual descriptions of these pre- to post-treatment changes for the treatment group specifically to the paragraph that describes the results for each respective variable.
11. We have added to each results paragraph that describes the primary results for each of the process variables – AAQ and VLQ – the results of preliminary associative analyses that are suggestive of the associations between the outcome and process variables (ie., Between AAQ and GAD, and between VLQ and depression and wellbeing). Pages 19 and 20
12. This limitation has been acknowledged and explained as suggested by the reviewer. page 25
13. We have added a paragraph that now acknowledges this aspect of the study’s design – its choice of a waitlist control, and its implications are further discussed. Pages 25 and 26.
